# Predictors of bleeding-related in-hospital complications after coronary angiography and percutaneous coronary intervention: A single-center retrospective cohort study in Iran

**DOI:** 10.1016/j.ijcrp.2026.200633

**Published:** 2026-04-08

**Authors:** Abbas Andishmand, Mohammad Hossein Soltani, Hamidreza Mohammadi, Seyedeh Mahdieh Namayandeh, Parisa Peigan, Matthew Budoff, Marzieh Azimizadeh, Mohsen Andishmand, Mojtaba Andishmand

**Affiliations:** a Yazd Cardiovascular Research Center, Non-communicable Diseases Research Institute, Shahid Sadoughi University of Medical Sciences, Yazd, Iran; b Department of Epidemiology, Shahid Sadoughi University of Medical Sciences, Yazd, Iran; c Lundquist Institute, Harbor-UCLA Medical Center, Torrance, CA, United States

**Keywords:** Percutaneous coronary intervention, Coronary angiography, Inguinal hematoma

## Abstract

**Background:**

Coronary angiography (CA) and percutaneous coronary intervention (PCI) are widely used for diagnosing and treating coronary artery disease (CAD) but may cause bleeding-related in-hospital complications, especially with femoral access. This study evaluated the incidence and predictors of access-site bleeding events and related outcomes in central Iran.

**Methods:**

In this retrospective cohort, 1369 patients underwent CA and PCI at Afshar Hospital, Yazd, between 2020 and 2022. Demographic, clinical, and procedural data were collected. Bleeding events were classified using the Bleeding Academic Research Consortium (BARC) criteria, and high bleeding risk was defined according to the Academic Research Consortium for High Bleeding Risk (ARC-HBR). Logistic regression identified independent predictors.

**Results:**

Bleeding-related in-hospital complications occurred in 143 patients (10.4%), most commonly inguinal hematoma (8.4%), major bleeding (1.6%), and mortality (0.6%). BARC Type 2 bleeding was most frequent (8.4%), followed by Type 3a (1.6%) and 3b (0.3%). Based on ARC-HBR, 13.5% of patients met at least one major or two minor high bleeding risk criteria. Multivariate analysis showed that elevated international normalized ratio (INR) (OR = 2.15; 95% CI: 1.22–3.72; P = 0.006) and anticoagulant use (OR = 1.8; 95% CI: 1.14–2.85; P = 0.011) were significantly associated with complications.

**Conclusion:**

Bleeding-related complications, particularly hematoma, major bleeding, and procedure-related mortality, occurred in over 10% of patients undergoing CA and PCI. Anticoagulant therapy and elevated INR were key predictors, highlighting the importance of individualized risk assessment and bleeding risk stratification using tools like BARC and ARC-HBR.

## 1 Introduction

Invasive coronary angiography (CA) is considered the gold standard for diagnosing coronary artery disease (CAD) [1]. Annually, over one million individuals in the United States undergo coronary angiography and percutaneous coronary intervention (PCI) [2]. Despite a growing preference for radial artery access, the femoral artery remains the most frequently used access site worldwide [3]. Access site complications are a significant contributor to morbidity and mortality following coronary procedures, with reported incidence rates of arterial access complications varying between 1% and 38% [[4], [5], [6]]. In Iran, the reported incidence of vascular complications following CA ranges from 0.9% to 24% [7,8].

Previous studies have reported that complications such as bleeding are associated with both short- and long-term adverse outcomes after PCI, often leading to prolonged hospital stays and increased healthcare costs [[9], [10], [11], [12]]. While enhancements in equipment quality, reduction in device profile, and advancements in operator skills and therapeutic methods are anticipated to reduce complication rates [13], bleeding complications remain a primary challenge in modern PCI despite a decline in their overall incidence [14,15].

However, the availability of data on bleeding complications following PCI within the Asian population is limited. Asian subgroups have been underrepresented in large-scale registries predominantly conducted in Western settings [16]. Cardiovascular risk factors differ across racial and ethnic groups, and Iranian patients may present unique profiles compared with Western populations. [[17], [18], [19]]. While numerous international studies have examined these risks, there is still limited evidence on in-hospital complications following PCI in Middle Eastern populations, particularly in Iran. Thus, efforts to reduce complications and precise risk prediction remain important. Based on these findings, the main objective of this study was to examine the incidence and risk factors for bleeding-related in-hospital complications in patients undergoing CA and PCI.

## 2 Methods

### 2.1 Study design and patient population

**Fig. 1:**
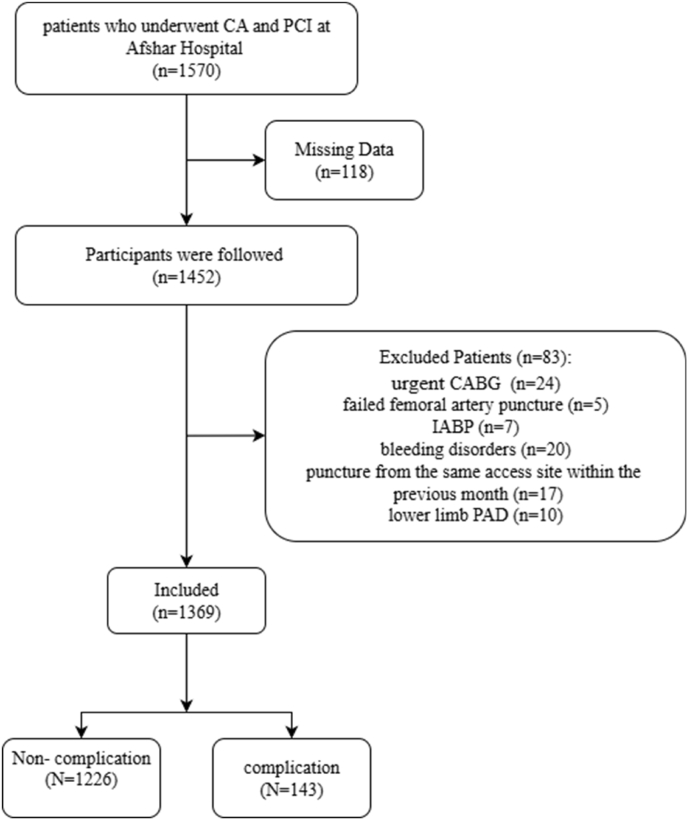
Flow chart of the study design.

This retrospective cohort study was conducted on 1369 patients who underwent both CA and PCI at Afshar Hospital affiliated to Shahid Sadoughi University of Medical Sciences (Yazd, Iran) from January 2020 to December 2022. This study was approved by the local ethics committee of Shahid Sadoughi University of Medical Sciences (IR.SSU.MEDICINE.REC.1398.276) and conducted based on the Declaration of Helsinki on medical research. Informed consent was obtained from eligible individuals. The patients who were at least 18 years of age were included. Exclusion criteria included urgent coronary artery bypass grafting (CABG) after CA, failed femoral artery puncture, concomitant femoral vein puncture, implementation of an intra-aortic balloon pump (IABP), bleeding disorders, puncture from the same access site within the previous month, and lower limb peripheral artery disease (PAD) (Fig. 1).

### 2.2 Coronary angiography and percutaneous coronary intervention

Interventional cardiologists performed procedures with a minimum of 5 years of experience. All patients underwent CA through the femoral artery. The right femoral artery access was the first choice, with the left femoral artery access as an alternative. The puncture site was located 2-3 cm below the inguinal ligament. An introducer needle (18G*7, SCW Meditech Company, Guangzhou, China) was used to enter the common femoral artery. If an arterial blood jet was detected, a j-tip guide wire with a diameter of 0.035 inches was passed through. Once confirmed to be inside the lumen, the arterial sheath (Avanti Plus, 6F, 11 cm, Cordis, Florida, USA) was inserted. CA was performed, and if necessary, PCI was also conducted. During PCI, an intravenous injection of 70-100 units of heparin per kilogram of body weight was administered, and the use of eptifibatide was optional based on the operator's clinical judgment.

### 2.3 Post-procedure

In the post-catheterization unit, all patients underwent non-invasive monitoring for hemodynamics and heart rhythm. The arterial sheath was removed, and hemostasis was performed. All patients were instructed to remain on complete bed rest for at least 6 h following sheath removal. The access site was checked repeatedly for the detection of complications. Initial management of vascular complications involved medical and conservative measures, including reapplying pressure to the access site, exchanging the existing sheath for one of a larger diameter, fluid infusion in case of hypotension, and injection of blood products in case of a drop in hemoglobin levels. Imaging modalities such as Doppler ultrasonography and CT angiography were used to detect the type and extent of vascular complications, or invasive aortoiliac angiography was used. Patients who did not respond to initial medical treatments were referred to a vascular surgeon or interventionalist for invasive intervention. Patients who underwent PCI received dual antiplatelet treatment (DAPT) with a maintenance dose including aspirin 80 mg and clopidogrel 75-150 mg or ticagrelor 90 mg twice daily. High-risk patients for thrombotic events received an intravenous (IV) infusion of heparin, eptifibatide, or both. Hemodynamically stable patients were monitored for 24 h, while unstable patients were monitored for 48 h or more. Patients with access site complications remained in the hospital until the complication improved [[20], [21], [22]].

### 2.4 Data collection

For all studied patients, a questionnaire was completed that included demographic information such as sex, age, and Body Mass Index (BMI). Charlson Comorbidity Index (CCI), diabetes Mellitus (DM), hypertension (HTN), dyslipidemia, family history of CAD, smoking, opium addiction, number of involved vessels, medications, laboratory tests, procedure status, and angiographic findings (Number of involved vessels). Data were collected through face-to-face interviews and a review of the patient's medical records. We defined the severity of co-morbid conditions using Deyo's modification of CCI [23]. The estimated glomerular filtration rate (eGFR) was calculated using the 2021 CKD-EPI creatinine equation, which does not include a race coefficient [24]. Data on complications from the hospital database were collated, and a cardiologist categorized the coronary procedural complications. The procedure-related complications were identified using the International Classification of Diseases, Ninth Revision, Clinical Modification (ICD-9-CM) codes. A hematoma was defined as a localized swelling ≥5 cm at the femoral access site due to bleeding under the skin, occurring during or after the procedure. Major bleeding was defined as life-threatening bleeding requiring transfusion of ≥2 units of packed red blood cells, or resulting in an absolute decrease in haematocrit of ≥10% or death, or haemorrhagic/subdural hematoma. All bleeding complications were classified using the Bleeding Academic Research Consortium (BARC) criteria [25]. High bleeding risk at baseline was assessed using the Academic Research Consortium for High Bleeding Risk (ARC-HBR) checklist [26].

### 2.5 Statistical analysis

Data were analyzed using SPSS 26 software (IBM SPSS Statistics 26, Chicago, IL, USA). Quantitative data were expressed as mean and standard deviation, while qualitative data were expressed as frequency and percentage. Independent T-tests were used to test quantitative variables, while Chi-square tests were used to test qualitative variables. Univariate and multivariate binary logistic regression were performed. Variables included in the multivariate regression model were selected based on clinical relevance, findings from prior literature, and significance in univariate analysis. The findings of these analyses were reported in terms of odds ratios (OR), along with corresponding 95% confidence intervals (95% CI). A two-sided P ≤ 0.05 was considered statistically significant.

### 2.6 Results

The mean age of the 1369 participants (828 Males) who underwent CA and PCI was 59.7 ± 11.4 years. Among a total of patients, 143 (10.4%) experienced one of the in-hospital complications; the most common complications were inguinal hematoma (8.4%), followed by major bleeding (1.6%) and mortality (0.6%). Among those with an inguinal hematoma, four patients also experienced major bleeding and required blood transfusion. In addition, six patients with hematoma concurrently developed a pseudoaneurysm (Table-1).

**Table-1:** In-hospital complications.

Complications	Frequency	Percentage
Overall complications	143	10.4
Inguinal hematoma	115	8.4
Major bleeding	22	1.6
Pseudoaneurysm	6	0.4
Mortality	8	0.6
Heart surgery	3	0.2
Postoperative DVT/PE	1	0.1
AV fistula	1	0.1

Abbreviations: DVT, Deep Vein Thrombosis; PE, Pulmonary Embolism; AV, arteriovenous.

Based on the BARC classification, the most common bleeding category among those affected was Type 2 (n = 115, 8.4%), followed by Type 3a (n = 22, 1.6%) and Type 3b (n = 4, 0.3%) (Table-2).

**Table-2:** Distribution of bleeding events according to the bleeding academic research consortium (BARC) classification.

	Frequency	Percentage
Type 0	1228	89.7
Type 2	115	8.4
Type 3a	22	1.6
Type 3b	4	0.3

The comparisons of demographic characteristics and the occurrence of comorbidities between the non-complication and complication groups did not differ significantly (P > 0.05). Risk factors such as emergent procedural status (P = 0.021) and number of involved vessels (P = 0.018) differed significantly between the groups. In the complications group, eGFR (P = 0.019) was significantly lower, and International normalized ratio (INR) (P = 0.001) and creatinine (P = 0.038) were significantly higher compared to the other group (Table-3). Mean INR was slightly higher in patients receiving oral anticoagulants (1.18 ± 0.29) compared to those not receiving anticoagulants (1.16 ± 0.25), but this difference was not statistically significant (p = 0.37). Additionally, the combined use of oral anticoagulant and single or double antiplatelet therapy showed no significant association with the occurrence of in-hospital complications (1.4 % vs. 0.7 %, P > 0.05).

**Table-3:** Baseline characteristics of study population.

Variable	Non- complication (N = 1226)	complication (N = 143)	Overall (N = 1369)	P-Value
Sex				0.52
Male	745 (60.8)	83 (58)	828 (60.5)	
Female	481 (39.2)	60 (42)	541 (39.5)	
Age, year	59.56 ± 11.3	61.13 ± 12.18	59.7 ± 11.4	0.12
≤50	271 (22.1)	25 (17.5)	296 (21.6)	0.029
50-60	377 (30.8)	50 (35)	427 (31.2)	
60-70	375 (30.6)	33 (23.1)	408 (29.8)	
70 ≤	203 (16.6)	35 (24.5)	238 (17.4)	
BMI, kg/m^2^	26.45 ± 4.3	26.57 ± 4.78	26.47 ± 4.37	0.79
Comorbidities				
CCI				0.38
0	174 (14.2)	16 (11.1)	190 (13.9)	
1	287 (23.4)	39 (27.3)	326 (23.8)	
2	382 (31.2)	38 (26.6)	420 (30.7)	
3 ≤	383 (31.2)	50 (35)	433 (31.6)	
Obesity	174 (14.2)	19 (13.3)	193 (14.1)	0.76
DM	446 (36.4)	46 (32.2)	492 (35.9)	0.32
HTN	611 (49.8)	72 (50.3)	683 (49.9)	0.9
Dyslipidemia	466 (38)	50 (32.2)	516 (37.7)	0.47
Family history of CAD	366 (29.9)	38 (26.6)	404 (29.5)	0.41
Current/recent smoking	194 (15.8)	20 (14)	214 (15.6)	0.56
Opium addiction	191 (15.6)	16 (11.2)	207 (15.1)	0.16
procedure status				0.021
Elective procedure	781 (63.7)	77 (53.8)	858 (62.7)	
Emergent procedure	445 (36.5)	66 (46.2)	511 (37.3)	
Number of involved vessels				0.018
SVD	582(47.5)	53 (37.1)	635 (46.4)	
MVD	644 (52.5)	90 (62.9)	734 (53.6)	
Antiplatelet drugs	827 (67.5)	89 (62.2)	916 (66.9)	0.21
P2Y12 inhibitor	563 (45.9)	57 (39.9)	620 (45.3)	0.16
Aspirin	757 (61.7)	77 (53.8)	834 (60.9)	0.067
Glycoprotein IIb/IIIa	1 (0.1)	1 (0.7)	2 (0.1)	0.067
Anticoagulant drugs	645 (52.7)	99 (69.2)	745 (54.4)	<0.001
Unfractionated heparin	628 (51.2)	95 (66.4)	723 (52.8)	<0.001
Low-Molecular-Weight Heparin	15 (1.2)	4 (2.8)	19 (1.4)	0.13
warfarin	14 (1.1)	3 (2.1)	17 (1.2)	0.32
Laboratory values				
Hemoglobin, g/dL	13.73 ± 1.71	13.71 ± 1.54	13.73 ± 1.69	0.87
Platelet count	243.7 ± 69.5	237.3 ± 56.4	243 ± 68.2	0.31
INR	1.16 ± 0.24	1.25 ± 0.46	1.17 ± 0.27	0.001
Creatinine, mg/dL	1.16 ± 0.42	1.25 ± 0.68	1.17 ± 0.25	0.038
eGFR, mL/min/1.73 m^2^	66.45 ± 17.88	62.49 ± 18.7	66.04 ± 18	0.019

Data presented as mean ± standard deviation or number (%). Abbreviations: BMI, Body Mass Index; CCI, Charlson Comorbidity Index; DM, Diabetes mellitus; HTN, Hypertension; CAD, Coronary artery disease; SVD, Single vessel disease; MVD, Multivessel disease; LMWH, Low-Molecular-Weight Heparin; INR, International normalized ratio.

According to ARC-HBR criteria, the most frequent major criterion was anticipated use of long-term oral anticoagulation (1.2%), followed by severe or end-stage CKD (2.2%) and baseline hemoglobin <11 g/dL (5.6%). Among the minor criteria, moderate CKD was most prevalent (30.9%), followed by hemoglobin 11–12.9 g/dL for men and 11–11.9 g/dL for women (14.5%), and age ≥75 years (9.9%). No statistically significant differences in the prevalence of criteria were observed between patients with and without in-hospital complications (all P > 0.05) (Table-4).

**Table 4:** Comparison of major and minor criteria of the Academic Research Consortium for High Bleeding Risk (ARC-HBR) between patients with and without in-hospital complications.

Variable	Non-complication (N = 1226)	complication (N = 143)	Overall (N = 1369)	P-Value
Major				
Anticipated use of long-term oral anticoagulation	14 (1.1)	3 (2.1)	17 (1.2)	0.32
Severe or end-stage CKD	26 (2.1)	4 (2.8)	30 (2.2)	0.6
Hemoglobin <11 g/dL	72 (5.9)	5 (3.8)	77 (5.6)	0.24
Minor				
Age ≥75 y	116 (9.5)	20 (14)	136 (9.9)	0.08
Moderate CKD	371 (30.3)	52 (36.4)	423 (30.9)	0.13
Hemoglobin 11–12.9 g/dL for men and 11–11.9 g/dL for women	178 (14.5)	21 (14.7)	199 (14.5)	0.95

**Fig. 2:**
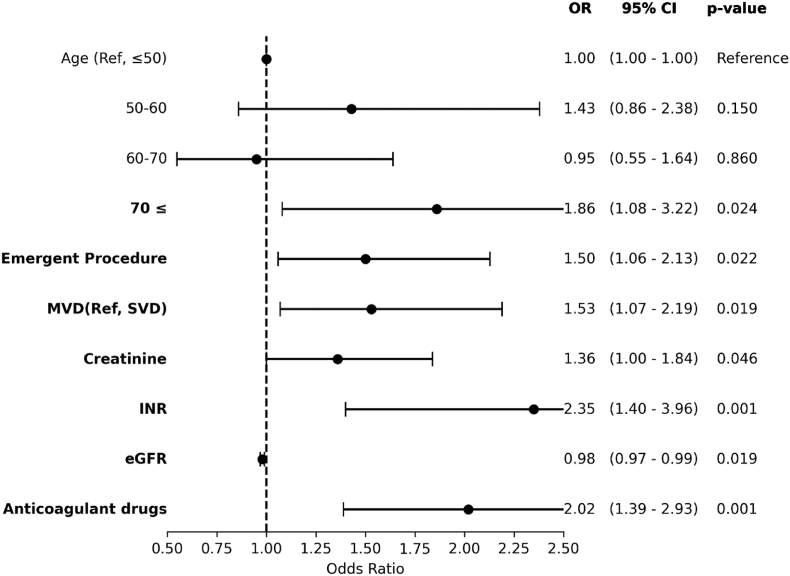
Univariate logistic regression of risk factors for in-hospital complications of PCI. The results are expressed as an odds ratio and 95% confidence intervals.

**Fig. 3:**
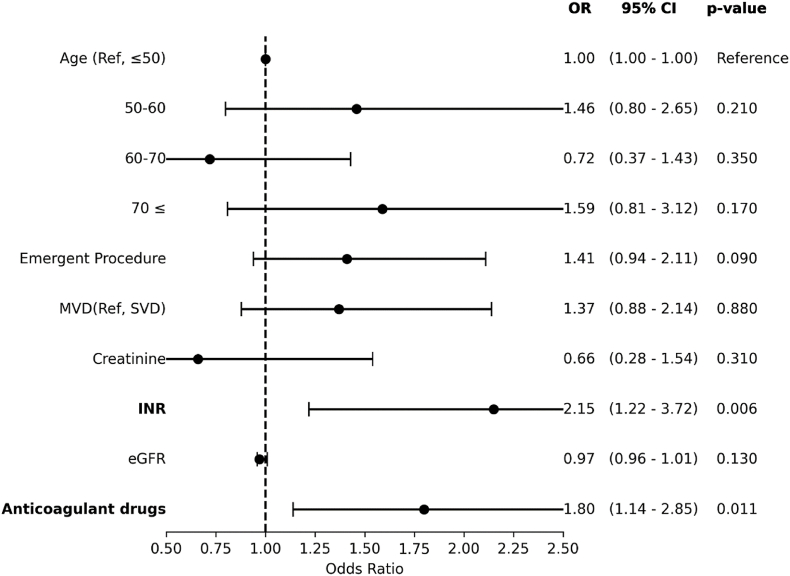
forest plot of final multivariable analysis for in-hospital complications of PCI. The results are expressed as an odds ratio and 95% confidence intervals.

Age≥70 years (OR:1.86, 95% CI: 1.08-3.2, P = 0.024), emergent procedural status (OR:1.5, 95% CI: 1.06-2.1, P = 0.022), multivessel disease (MVD) (OR:1.53, 95% CI: 1.07-2.2, P = 0.019), elevated INR (OR:2.35, 95% CI: 1.4-3.9, P = 0.001), and anticoagulant drug use (OR:2.02, 95% CI: 1.4-2.93, P = 0.001) were identified as significant risk factors in the univariate analysis (Figure-2). However, in the multivariate analysis anticoagulant drug use (OR = 1.8, 95% CI: 1.14-2.85, P = 0.011) and elevated INR (OR: 2.15, 95% CI: 1.22-3.72, P = 0.006) were significantly associated with a higher risk of in-hospital complications after adjustment for other variables (Figure-3).

## 3 Discussion

This study reports the incidence and predictors of bleeding-related in-hospital complications after CA and PCI in a population from central Iran. Common complications reported in this study were inguinal hematoma, major bleeding, pseudoaneurysm, and mortality. A total of 10.4% of patients experienced complications during their hospital stay. Other studies have reported complication rates after PCI ranging from 1.6% to 9.5% [[27], [28], [29]]. While our rate is higher than that observed in these studies, it is important to recognize that one factor contributing to the increased incidence of in-hospital complications after PCI is the variation in how complications are classified and recognized across different studies. For instance, in the study by Arora et al., inguinal hematoma was not considered a complication of PCI [30].

Furthermore, the differences in complication rates across studies can be attributed to several factors, including patient characteristics, the type of procedure performed, the equipment used, the patient's overall clinical condition, the operator's expertise, and the quality of post-procedure care. Unlike studies that broadly assess ischemic, contrast-induced, or systemic complications, our analysis was confined to bleeding-related outcomes and associated mortality. This focused approach provides valuable insight into predictors of femoral access bleeding complications and related mortality, which remain clinically significant despite advances in PCI techniques.

Complications following PCI are associated with the occurrence of major cardiovascular events (MACE) in both the short and long term [31]. Therefore, recognizing predictive factors for PCI complications can significantly enhance patient outcomes. In this regard, we investigated the impact of demographic factors, comorbidities, angiographic findings, and medication use on the occurrence of PCI complications.

Although studies show that elderly patients have a higher risk for getting complications following CA and PCI [[32], [33], [34]], our analysis did not find age to be an independent predictor of complications when other factors were adjusted. Moreover, in most studies, female have been identified as a significant risk factor for increased complications after PCI [35,36]. Smaller vessel size, a higher risk profile with a higher burden of comorbidities, have been proposed to explain the higher rate of complications following PCI [37,38]. However, we observed conflicting results. Similarly, Abtan et al. found that sex was not associated with complications following PCI [39]. This topic remains controversial, and it is essential to conduct studies with larger sample sizes. Additionally, the simultaneous effect of other risk factors needs to be examined.

Previous studies have shown a higher rate of complications in patients with a higher burden of comorbidities [40,41]. However, the CCI and the difference in prevalence of comorbidities between groups were not statistically significant in the present study. This lack of significance may be due to the relatively high baseline level of comorbidities in our study population.

Various studies have shown that emergency procedures are independent predictors of increased vascular complications [42,43]. Additionally, procedural complexity, which may involve MVD or emergent procedures, can increase the likelihood of complications. These challenging procedures often require longer durations and higher volumes of contrast media, which can elevate the risk of vascular complications, acute kidney injury, and bleeding. Furthermore, operator experience and the number of procedures performed are critical factors in reducing these risks. Less experienced operators may inadvertently contribute to higher complication rates [44]. Estrada et al. indicated that higher procedural complexity, such as MVD, was associated with increased rates of both access site complications and MACE [45]. Panaich et al. found that patients with MVD had a higher rate of in-hospital complications, though mortality rates were similar between the groups [46]. Contrary to these studies, Omer et al. found that MVD was associated with a lower rate of in-hospital mortality and concluded that there is no definitive finding on this topic until further studies are conducted [47]. Our results align with Numasawa et al., who reported elevated INR as a bleeding predictor, but differ from Omer et al. regarding MVD and mortality.

Antiplatelet and anticoagulation therapy are critical in reducing rates of mortality, adverse ischemic events, short- and long-term complications of PCI, and other MACEs [[48], [49], [50]]. Despite their proven benefits, the use of these agents generally increases the risk of bleeding events. Steven et al. stated that newer and more potent agents and regimens may further increase the risk of bleeding, which will likely decrease compliance and increase the rates of cessation of therapy [51]. Nakagawa et al. reported that the efficacy of glycoprotein IIb/IIIa inhibitors in preventing post-PCI coronary events in Japanese patients was not detected [52]. Abtan et al. also reported that glycoprotein IIb/IIIa inhibitors are likely associated with an increased risk of complications following PCI [39]. Paradoxically, the benefit of these agents may be offset by this increase in bleeding. Variations in bleeding complication rates are often attributed to the different definitions and use of antiplatelets and anticoagulants across various studies [53]. In this context, we observed that the use of anticoagulant medications such as heparin was associated with an increase in complications after PCI. This issue may be attributed to improper medication use [54,55]. Also, Robert et al. found that a decrease in heparin usage correlates with a reduction in complications following PCI and CA [56]. Andersen et al. found that anticoagulant therapy increases the risk of complications after CA and PCI [57]. As Numasawa et al. have stated, an appropriate dosage of antiplatelets or anticoagulants in individual cases may help to reduce bleeding complications; the selection of antiplatelets and anticoagulants should be based on the individual's risk for both ischemic and bleeding complications after PCI [29]. Our finding highlights the importance of personalized patient evaluations before any intervention to tailor procedural strategies and optimize post-procedure care, ultimately improving patient outcomes by reducing in-hospital complications [58].

Consistent with other studies, the present study found that elevated INR levels were associated with a higher likelihood of in-hospital complications following PCI [59]. This higher INR may be attributed to several factors, including increased use of anticoagulant therapies, as a higher percentage of patients in the complication group were receiving anticoagulants. Secemsky et al. found that patients on chronic oral anticoagulation undergoing PCI have a higher risk of in-hospital bleeding compared to those not on anticoagulation therapy [60]. The potential impact of kidney function should also be considered; patients with impaired renal function, who often have higher baseline INR values, may experience an amplified response to anticoagulants. Several studies demonstrated that patients with lower eGFR have a greater INR, which significantly increases the risk of hemorrhage [61,62]. Additionally, renal impairment was associated with increased risks of thrombotic and bleeding complications in patients receiving higher doses of anticoagulant medications [63].

Additionally, comorbidities and age, common among patients with complications, might further heighten their sensitivity to anticoagulation therapy. In this regard, Rouaud et al. found that low-quality control of INR is associated with a CCI of 3 or greater in elderly patients [64]. Another study showed that older patients are more likely to experience over-anticoagulation during induction and have higher INR values in the maintenance phase of warfarin therapy [65]. These findings underscore the importance of careful monitoring and individualized dosing of anticoagulants in patients undergoing PCI, particularly among those with complex clinical profiles.

In the present study, the majority of patients experienced no bleeding (BARC 0: 89.7%), and major bleeding events (BARC 3a and 3b) were rare (1.9%). Furthermore, no statistically significant differences were found in ARC-HBR major or minor criteria between patients with and without in-hospital complications. This pattern aligns with data from the VCOR registry in Australia, where major in-hospital bleeding (BARC 3–5) was observed in less than 1% of PCI patients, while minor bleeding (BARC 1–2) occurred in 7.9% [66]. The slightly higher BARC 2 rate in our cohort (8.4%) may reflect population differences, higher-risk presentations (e.g., ACS), or reporting variability.

Our finding of low rates of major bleeding is consistent with previous reports. For example, Matić et al. showed that although major bleeding events (BARC ≥3) following primary PCI were uncommon, they significantly impacted both short- and long-term outcomes [67].

Regarding bleeding risk prediction, the ARC-HBR criteria did not differentiate between patients with and without in-hospital complications. This is not unexpected, as the ARC-HBR definition was developed to identify patients at high 1-year risk of major bleeding (i.e., BARC 3 or 5 ≥ 4%) rather than to predict in-hospital events [26]. In validation studies from the Bern PCI registry, ARC-HBR patients had significantly higher 1-year major bleeding rates (6.4% vs. 1.9%), and the ARC-HBR score outperformed PRECISE-DAPT and PARIS scores in long-term bleeding prediction [68,69].

### 3.1 Limitations

This study has several limitations. First, its retrospective design may introduce selection bias and limit control over data collection, as some clinical information may not have been consistently documented. Second, the short follow-up period likely underestimated the incidence of late-onset complications, such as pseudoaneurysm and arteriovenous fistula, which could have been missed post-discharge. Third, detailed anatomical information was not collected, and therefore the Gensini and Taxus and Cardiac Surgery (SYNTAX) scores to predict in-hospital complications of PCI could not be evaluated. Furthermore, although all patients underwent both diagnostic coronary angiography and PCI, the exact post-procedural dosage and duration of unfractionated heparin or eptifibatide and the activated partial thromboplastin time (aPTT) were not consistently recorded and therefore could not be analyzed. Lastly, the study exclusively focused on femoral arterial access, which limits the applicability of the results to patients who undergo PCI or CA through radial artery access.

## 4 Conclusion

In participants who underwent CA and PCI, 10.4% experienced in-hospital complications. The most common complications were inguinal hematoma, followed by major bleeding and mortality. Factors such as emergent procedural status, MVD, elevated INR, and anticoagulant drug use were identified as significant predictors of complications. A comprehensive understanding of these factors can guide clinical decision-making and improve patient safety. Given the significant morbidity associated with femoral access site complications, particularly bleeding and hematoma, future research should focus on strategies to further reduce these risks, including the consideration of alternative access routes such as radial artery access in suitable patients. Additionally, prospective studies with extended follow-up periods would provide a more comprehensive understanding of the long-term outcomes of access site complications.

## CRediT authorship contribution statement

**Abbas Andishmand:** Writing – review & editing, Supervision, Project administration, Data curation. **Mohammad Hossein Soltani:** Writing – review & editing, Writing – original draft, Software, Methodology, Formal analysis, Conceptualization. **Hamidreza Mohammadi:** Writing – review & editing, Writing – original draft, Validation, Project administration, Methodology, Investigation, Data curation. **Seyedeh Mahdieh Namayandeh:** Validation, Supervision, Methodology, Data curation, Conceptualization. **Parisa Peigan:** Writing – review & editing, Writing – original draft, Validation, Data curation. **Matthew Budoff:** Writing – review & editing, Validation, Investigation. **Marzieh Azimizadeh:** Writing – review & editing, Writing – original draft, Supervision, Methodology. **Mohsen Andishmand:** Writing – review & editing, Writing – original draft, Conceptualization. **Mojtaba Andishmand:** Writing – review & editing, Writing – original draft, Formal analysis, Data curation.

## Ethics approval and consent to participate

This study was approved by the local ethics committee of Shahid Sadoughi University of Medical Sciences (IR.SSU.MEDICINE.REC.1398.276) and conducted based on the Declaration of Helsinki on medical research. Informed consent was obtained from eligible individuals.

## Consent for publication

All participants have signed an informed consent, which includes the consent for publication.

## Availability of data and materials

The datasets used and/or analysed during the current study are available from the corresponding author on reasonable request.

## Transparency statement

The lead author, Abbas Andishmand, affirms that this manuscript is an honest, accurate, and transparent account of the study being reported; that no important aspects of the study have been omitted; and that any discrepancies from the study as planned (and, if relevant, registered) have been explained.

## Funding

None.

## Declaration of competing interest

All authors declare that there was no conflict of interest for the present study.
